# Rectal Culture-Guided Targeted Antimicrobial Prophylaxis Reduces the Incidence of Post-Operative Infectious Complications in Men at High Risk for Infections Submitted to Transrectal Ultrasound Prostate Biopsy – Results of a Cross-Sectional Study

**DOI:** 10.1371/journal.pone.0170319

**Published:** 2017-01-25

**Authors:** Luca Boeri, Matteo Fontana, Andrea Gallioli, Stefano Paolo Zanetti, Michele Catellani, Fabrizio Longo, Barbara Mangiarotti, Emanuele Montanari

**Affiliations:** 1 Department of Urology, Fondazione IRCCS Ca' Granda - Ospedale Maggiore Policlinico, University of Milan, Milan, Italy; 2 Department of Urology, Ospedale San Paolo, University of Milan, Milan, Italy; University of British Columbia, CANADA

## Abstract

The role of rectal culture-guided antimicrobial prophylaxis (TAP) in reducing infectious complications (IC) after transrectal-ultrasound prostate biopsy (TRUSPBx) is conflicting. We assessed the prevalence of IC in a cohort of men at high risk for IC submitted to TRUSPBx and treated with either TAP or empirical prophylaxis (EAP). Data from 53 patients at high risk for IC undergoing TRUSPBx were collected. Patients who did not receive a rectal swab (RS) were treated with EAP with fluoroquinolones (FQs). Of those who received the RS, patients with FQ-susceptible organisms received ciprofloxacin while those with FQ-resistant organisms received TAP. Office visits were scheduled to investigate the rate of complication at day 7 and 30 after TRUSPBx. Comorbidities were scored with the Charlson Comorbidity Index (CCI). Descriptive statistics and logistic regression models detailed the association between clinical parameters and IC rate. Out of 53 men, 17 (32.1%) had RS while 36 (67.9%) did not. All RS cultures were positive for E. Coli and 4 (23.5%) reported FQ-resistant pathogens. Considering risk factors for IC, no difference was found in terms of CCI, rate of diabetes, UTIs or recent antibiotic utilization between groups. Overall, 12 (22.6%) men reported IC, with a greater proportion of them belonging to the group treated with EAP (30.6% vs 5.9%; p = 0.045). Of these, 9 (25.0%) patients, all treated with EAP, developed post biopsy UTIs. E. Coli sustained all UTIs and 7 (77.7%) were FQ resistant. At multivariable analysis, CCI≥1, a history of UTIs/prostatitis and recent antibiotic utilization (all p<0.04) were the most powerful predictors for ICs. In conclusion, we found that compared to EAP, TAP significantly reduces ICs, in men at high risk for post TRUSPBx IC. Patients at risk for IC, especially those with recent antibiotic utilization, CCI≥1 and a history of UTIs/prostatitis before biopsy, could benefit from TAP.

## Introduction

Prostate cancer (PCa) is the most common non-skin cancer in elderly males in Europe. Transrectal ultrasound-guided prostate biopsy (TRUSPBx) is currently the standard tissue-sampling technique for the histological diagnosis of PCa, with over one million patients undergoing biopsy each year in the United States [1]. Even if TRUSPBx is generally considered a safe procedure, it may be accompanied by clinical complications ranging from pain, haematospermia and haematuria to severe infectious complications such as urinary tract infections (UTIs), prostatitis and sepsis [2]. Studies have shown high rates of post TRUSPBx infectious complications (PTICs), with evidence of an increasing trend [2,3]. *Escherichia Coli* (E. coli) is the most commonly implicated pathogen in PTICs, present in 75–90% of cases [4].

Antimicrobial prophylaxis has shown high efficacy in reducing PTICs [5] and fluoroquinolones (FQs) are the current first-line recommended antibiotics. However, infectious complications due to FQ-resistant organisms have increased in recent years [6,7] and FQ-resistant organisms are becoming a significant health problem. Indeed, pre-biopsy rectal cultures have demonstrated a FQ-resistant colonization rate of 10% to 22% [8,9].

The first step for PTIC prevention is a preoperative assessment of risk factors for resistant bacteria [10]. Risk factors include the exposure to antimicrobials within six months prior to biopsy and the presence of FQ-resistant bacteria [9,11]. Hospital employees and international travellers to areas where FQ resistance is endemic are also at increased risk for infection [12,13]. Moreover, prior prostate biopsy may be another risk factor for infectious complications, though this claim has been disputed [14].

Rectal swab (RS) cultures are used to determine the presence of FQ-resistant flora prior to TRUSPBx [15,16]. Previous studies investigating the role of RS-guided targeted antimicrobial prophylaxis (TAP) before TRUSPBx in reducing PTICs have found similar but not unanimous results [17–19]. Importantly, these studies applied TAP to all patients, regardless of pre-existing risk factors for PTICs.

Concerns exist regarding the necessity, logistics, and cost-effectiveness of performing RS cultures in all patients, but a selected approach for men with known risk factors may have benefits [16]. To the best of our knowledge, no prospective studies have evaluated the effectiveness of RS-guided prophylaxis in preselected at-risk populations. To address this gap, we performed a cross-sectional study evaluating the effectiveness of TAP in reducing infectious complications after TRUSPBx in a selected cohort of patients at high risk for PTICs.

## Materials and Methods

### Patients

**Fig 1:**
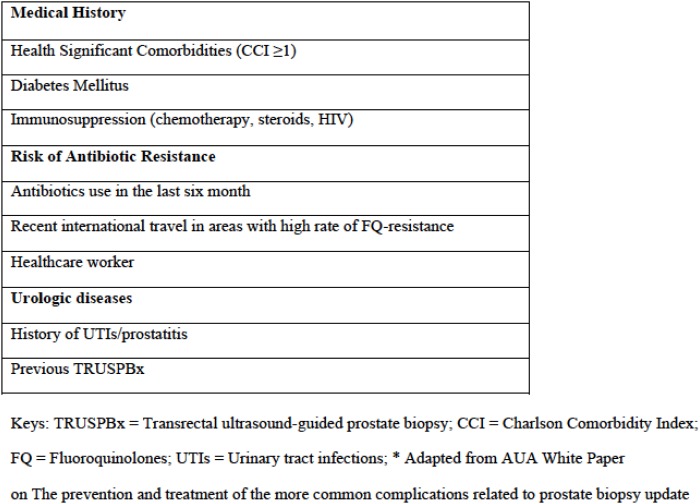
Risk factors for post TRUSPBx infectious complications.

The analyses were based on retrospective data from a cohort of 53 consecutive white-European individuals submitted to TRUSPBx for the suspicion of PCa between October 2015 and May 2016 at a single academic clinic. An additional 7 patients were enrolled in the study but ultimately excluded due to incomplete data collection. Patients seen between October 2015 and February 2016 underwent a RS culture and received TAP based on results, as per the standard of care during that period. Patients seen between March 2016 and May 2016 received EAP (500mg ciprofloxacin 2 hours before and 12 hours after the procedure) without undergoing a rectal swab, according to a change in the standard protocol of the clinic. Each individual patients were enrolled only once during the course of the study. Inclusion criteria were: the presence of one or more known risk factors for PTICs [10] (Fig 1) and the absence of UTIs at the time of biopsy. Pre biopsy UTIs were documented by culture. A history of travel was defined as international travel within six months prior to biopsy. Antimicrobial use was defined as oral or intravenous antimicrobial therapy (specifically fluoroquinolones, first-, second-, and third-generation cephalosporins and aminoglycoside) within the six months preceding biopsy (taken for at least seven days continuatively).

A detailed medical history was collected. Health-significant comorbidities were scored with the Charlson Comorbidity Index [20] as either continuous or categorized variables (CCI; categorized 0 vs ≥1). Body mass index (BMI) was also considered for each patient using the cut-offs proposed by the National Institutes of Health. Rectal swabs were collected in the clinic 10 days before the biopsy to allow adequate time for results. No patients refused to undergo the indicated RS. For FQ resistance testing, swabs were directly cultured on both MacConkey agar with 1 μg/mL ciprofloxacin and on blood agar plates and incubated at 35°C for 48 hours. Antibiotic susceptibilities were determined using the automated Vitek2 machine. Growth on both regular MacConkey and MacConkey with ciprofloxacin indicated presence of FQ-resistant Enterobacteriaceae species. All FQ-resistant isolates were further characterized (identification and antimicrobial susceptibilities on the Vitek-2 using GN and AST-GN25 cards). The combination MacConkey and MacConkey with ciprofloxacin plates detected 100% of Enterobacteriaceae isolates that tested intermediate or resistant to ciprofloxacin (MIC ≥2ug/mL) on the Vitek2, including ESBL-producing isolates.

Patients with FQ-sensitive microorganisms isolated by RS received FQs prophylaxis based on the EAP protocol, while those with FQ-resistant organisms received a TAP, for which the isolates were susceptible, developed by Infectious Diseases physicians. All patients complied with the prescribed prophylactic therapy. All patients received a Fleet enema the night before the procedure. All patients underwent dipstick urinalysis the same day of the procedure, before TRUSPBx to confirm nitrite negative, leukocyte esterase-negative urine. In case of positive results, patients were excluded from the study. All biopsies were performed by the same experienced urologist (with an experience of more than 100 biopsies) in an outpatient setting. Before TRUSPBx, local anaesthesia was administered transrectally. Prostate biopsies (18 cores) were taken using an automated biopsy gun with a disposable 18 gauge x 30cm biopsy needle. When clinically indicated (in the case of metastatic prostate cancer or saturation biopsy), 24 biopsy cores were taken. All patients were instructed to return to the emergency department of the same hospital if they developed post-biopsy complications. Urine culture was performed 7 days after the procedure. Follow-up visits for the evaluation of possible complications were scheduled 7 and 30 days after TRUSPBx as per standard clinic protocol. Complications were divided into two groups. Minor complications were defined as expected side effects of TRUSPBx, causing minimal or no discomfort, and requiring no additional treatment. Major complications were defined as adverse effects causing significant discomfort, disability, or requiring additional treatment. PTICs included postoperative fever >38°C, UTIs, prostatitis, bacteremia and sepsis. Symptomatic UTIs were defined according to EAU guidelines [21]. Prostatitis were clinically defined as frequent urge to urinate, difficulty urinating, chills and fever, pain the genitalia/pelvic area and painful digital rectal examination.

### Ethical approval

Data collection was carried out following the principles outlined in the Declaration of Helsinki; after being approved by the San Paolo Hospital Ethical Committee, all patients signed an informed consent agreeing to supply their own anonymous data for this and future studies.

### Statistical analyses

Data are presented as means (SD; ranges). The statistical significance of differences in means and proportions was tested with the one-way analysis of variance (ANOVA) and Pearson chi-square test, respectively. A 95% confidence interval was estimated for the association of categorical parameters. Exploratory analyses were initially applied to all variables; variables were retained for analysis when deemed clinically significant to the results. We performed logistic regression analysis on the cohort of men who received EAP to determinate the most powerful predictor of PTICs among the well-known risk factors (Fig 1) reported in our cohort of men. Logistic regression univariable analysis (UVA) and multivariable analysis (MVA) tested the associations between predictors and PTICs. Statistical analyses were performed using SPSS statistical software, v 13.0 (IBM Cor., Armonk, NY, USA). All tests were two sided, with a significance level set at 0.05.

## Results

Complete data collection was available for 17 (32.1%) patients who received RS and 36 (67.9%) who did not received RS (-RS) before TRUSPBx.

Table 1 details patient characteristics and descriptive statistics of the entire cohort. No differences in terms of age, BMI and mean PSA value were observed between groups. Overall, PCa was found in 32 (60.4%) individuals with no difference between groups. All RS cultures were positive for E. Coli and 4 (23.5%) contained FQ-resistant pathogens. Patients with FQ resistance received Trimethoprim-Sulfamethoxazole 800+160 mg 2 h before and 6 h after the procedure.

**Table 1:** Characteristics and descriptive statistics of patients (No. = 53).

	Overall	Rectal Swab	Non-swab	p value (F)*
No. of patients [No. (%)]	53 (100)	17 (32.1)	36 (67.9)	
Age (years)				0.36 (0.83)
Mean (SD)	69.2 (7.2)	70.5 (4.4)	68.6 (8.1)	
Range	54–82	62–81	54–82	
Categorized age [No. (%)]				0.13 (χ^2^, 5.56)
50–60	8 (15.1)	0 (0.0)	8 (15.1)	
61–70	23 (43.4)	10 (18.9)	43 (5.2)	
71–80	17 (32.1)	6 (11.3)	11 (20.8)	
≥81	5 (9.4)	1 (1.9)	4 (7.5)	
BMI (kg/m^2^)				0.23 (1.46)
Mean (SD)	25.3 (3.0)	26.0 (3.1)	24.9 (3.0)	
Range	17.8–33.1	20.5–33.1	17.8–31.1	
Categorized BMI [No. (%)]				0.51 (χ^2^, 1.32)
18.5–24.9	24 (45.3)	6 (35.1)	18 (50.0)	
25–29.9	25 (47.2)	9 (52.9)	16 (44.4)	
≥30	4 (7.5)	2 (11.8)	2 (5.6)	
PSA (ng/ml)				0.41 (0.69)
Mean (SD)	13.1 (19.7)	16.4 (29.7)	11.6 (12.9)	
Range	1.9–130.0	4.3–130.0	1.9–68.1	
Prostate Volume (ml)				0.82 (0.05)
Mean (SD)	63.2 (38.7)	64.9 (27.7)	62.3 (43.3)	
Range	14.0–234.0	27.0–120.0	14.0–234.0	
Prostate Adenoma (ml)				0.69 (0.16)
Mean (SD)	31.7 (24.4)	33.7 (18.7)	30.8 (26.9)	
Range	5.0–143.0	12.0–66.0	5.0–143.0	
Biopsy results [No. (%)]				0.66 (χ^2^, 0.19)
PCa	32 (60.4)	11 (64.7)	21 (58.3)	
Benign	21 (39.6)	6 (35.3)	15 (41.7)	
Biopsy Gleason Score [No. (%)]				0.32 (χ^2^, 1.97)
6	17 (53.1)	4 (36.4)	13 (61.9)	
7	8 (25.0)	4 (36.4)	4 (19.0)	
≥8	7 (21.9)	3 (27.3)	4 (19.0)	
Biopsy cores [No. (%)]				0.13 (χ^2^, 2.32)
18	46 (86.8)	13 (76.5)	33 (91.7)	
24	7 (13.2)	4 (23.5)	3 (8.3)	

BMI = body mass index; PSA = Prostate Specific Antigen; PCa = Prostate Cancer;

*P value according to chi-square test or analysis of variance (ANOVA), as indicated

Table 2 lists descriptive statistics in terms of risk factors for infectious complications and post TRUSPBx complication rates for the entire cohort of men. CCI scores were similar for the TAP vs EAP group (p = 0.08). Likewise, no differences in rates of diabetes mellitus, healthcare workers or a history of previous TRUSPBx were seen between groups. Out of 53 patients, 16 (30.2%) and 19 (35.8%) men reported antibiotic utilization and a history of UTIs/prostatitis in the 6 months preceding biopsy, respectively, with no difference between groups. None of the men included in the study reported recent travel to locations known for high rates of FQ-resistance or a positive anamnesis for immunosuppression (e.g steroids, chemotherapy, HIV).

**Table 2:** Characteristics and descriptive statistics [No. (%)] of patients (No. = 53).

	Overall		Rectal Swab (N. = 17)	Non-swab (N. = 36)	p value (χ^2^)*
Risk factors for infectious complications					
CCI					0.08 (F = 3.24)
Mean (SD)		1.1 (1.2)	1.3 (1.1)	0.6 (1.2)	
Range		0.0–6.0	0.0–3.0	0.0–6.0	
CCI					0.17 (1.85)
CCI 0		29 (54.7)	7 (41.2)	22 (61.1)	
CCI ≥1		24 (45.3)	10 (58.8)	14 (38.9)	
Diabetes Mellitus		5 (9.4)	3 (17.6)	2 (5.6)	0.16 (1.97)
UTIs/Prostatitis		16 (30.2)	6 (35.3)	10 (27.8)	0.58 (0.31)
Previous TRUSPBx		8 (15.1)	4 (23.5)	4 (11.1)	0.24 (1.38)
Healthcare workers		9 (17.0)	4 (23.5)	5 (13.9)	0.38 (0.76)
Recent antibiotic use		19 (35.8)	8 (47.1)	11 (30.6)	0.24 (1.36)
Post TRUSPBx minor complications					
Haematuria		14 (26.4)	4 (23.5)	10 (27.8)	0.74 (0.11)
Rectal bleeding		2 (3.8)	0 (0.0)	2 (5.6)	0.32 (0.98)
Hematospermia		18 (34.0)	5 (29.4)	13 (36.1)	0.63 (0.23)
Post TRUSPBx major complications					
Pain		4 (7.5)	2 (11.8)	2 (5.6)	0.42 (0.63)
Urinary retention		3 (5.6)	0 (0.0)	3 (8.3)	0.22 (1.50)
Fever > 38°C		13 (24.5)	1 (5.9)	12 (33.3)	0.03 (4.70)
Infectious complications		12 (22.6)	1 (5.9)	11 (30.6)	0.04 (4.02)
UTIs		9 (17.0)	0 (0.0)	9 (25.0)	0.02 (5.12)
Prostatitis		3 (5.6)	1 (5.9)	2 (5.6)	0.96 (0.02)
Hospitalization		4 (7.5)	0 (0.0)	4 (11.1)	0.15 (2.04)

CCI = Charlson Comorbidity Index; UTIs = Urinary tract infections; TRUSPBx = Transrectal ultrasound-guided prostate biopsy

*P value according to chi-square test or analysis of variance (ANOVA), as indicated

Hematospermia (18/53; 34%) and haematuria (14/53; 26.4%) were the most prevalent post TRUSPBx minor complications among patients. No differences in terms of minor complications were seen between groups. Overall, pain and urinary retention after biopsy were reported by only 4 (7.5%) and 3 (5.6%) patients. Post biopsy fever was more frequently reported by—RS patients (33.3% vs 5.9%; p = 0.03). Overall, 12 (22.6%) men reported PTICs, with a greater proportion of them belonging to the—RS group (30.6% vs 5.9%; p = 0.045). Of these, 9 (25.0%) patients, all belonging to the—RS group, developed post biopsy UTIs. E. coli sustained all UTIs and 7 (77.7%) were FQ resistant. According to guidelines, patients who developed PTICs were treated with third-generation cephalosporins, based on antimicrobial susceptibility. Three patients developed prostatitis after the procedure (2 of them sustained by E. coli and 1 sustained by Klebsiella sp.) and were treated with third-generation cephalosporins. Only 4 (7.5%) patients required hospitalization for PTICs with no differences between groups. No cases of sepsis occurred after biopsy.

Table 3 reports UVA and MVA analyses assessing the association between predictors and PTICs.

**Table 3:** Logistic regression models predicting post TRUSPBx infectious complications in patients who were not submitted to rectal swab (n = 36).

	Infectious complications			
	UVA model		MVA model	
	OR (95% CI)	P value	OR (95% CI)	P value
CCI≥1	4.31 (1.10–6.93)	0.03	7.08 (1.02–9.43)	0.04
Diabetes Mellitus	0.84 (0.08–8.823	0.88	0.31 (0.01–7.70)	0.48
+UTIs/Prostatitis	4.97 (1.27–9.50)	0.02	6.13 (1.04–6.32)	0.04
Previous TRUSPBx (Yes vs no)	0.44 (0.05–3.99)	0.46	0.14 (0.01–2.63)	0.19
Healthcare workers	0.97 (0.17–5.43)	0.97	0.25 (0.03–2.40)	0.23
Recent antibiotic use	4.34 (1.12–6.75)	0.03	5.53 (1.43–7.54)	0.02

UVA = Univariate model; MVA = Multivariate model, TRUSPBx = Transrectal ultrasound-guided prostate biopsy; CCI = Charlson Comorbidity Index; UTIs = Urinary tract infections

According to UVA, CCI≥1, a history of UTIs/prostatitis and recent antibiotic utilization were significantly associated with PTICs (all p<0.05). At MVA, CCI≥1 (OR 7.08, p = 0.04), a history of UTIs/prostatitis (OR 6.13, p = 0.04) and recent antibiotic utilization (OR 5.53, p = 0.02) were the only independent predictors for PTICs, after accounting for diabetes mellitus, being a healthcare worker and a history of previous TRUSPBx.

## Discussion

This study was designed to evaluate PTIC prevalence in a selected cohort of men at high risk for infectious complications after prostate biopsy who received a RS culture and subsequent TAP, compared to a group of same-risk men not receiving RS and treated with EAP before TRUSPBx. Of clinical importance, patients treated with EAP reported higher rates of PTICs compared to those treated with TAP. Importantly, 23.5% of patients with RS culture results were found to have FQ-resistant E. coli. Moreover, recent antibiotics utilization, CCI≥1 and a history of UTIs/prostatitis before biopsy were the most powerful predictors for PTICs.

Our interest was fuelled by increasing evidence regarding the role of RS-guided prophylaxis in reducing PTICs. Given the increasing trend of infectious complications due to FQ-resistant organisms [6,7], antimicrobial prophylaxis using RS culture shows promise to deliver several advantages. However, the benefits of TAP and its impact on PTICs, in comparison with EAP, are still debated. One study looking at this issue found no differences between men treated with TAP and those treated with EAP [18], others have shown that TAP results in fewer infectious complications than EAP yet with differences that failed to reach significance [22–24], while yet others [17,25] did find significant differences in the incidence of PTICs between men receiving TAP and patients treated with EAP prior to biopsy. More recently, a systemic review of the literature reported that PTIC and sepsis rates were significantly higher in men receiving EAP, compared to those treated with RS-guided TAP [19]. Overall, although multiple studies have found a reduction of PTICs when RS and TAP are used in the general population of men undergoing TRUSPBx, this difference has not always reached significance.

To the best of our knowledge, no studies have investigated the benefit of RS-guided TAP in a cohort of patients specifically selected based on their high risk for PTICs. We found that high-risk patients treated with EAP more frequently reported PTICs, compared to those treated with TAP. Furthermore, 23.5% of the RS cultures revealed FQ-resistant E. coli. This data is in line with previous studies showing increasing rates of PTICs owing to FQ-resistant bacteria [16], with rates of FQ-resistant E. coli ranging from 13% to 22% [16]. This finding is of primary importance because statistical models predicted that by 2013 the rate of FQ-resistant E. coli would have been as high as 45% in populations with high FQ usage [26]. Nevertheless, FQs are still the most commonly prescribed antibiotic prophylaxis prior to TRUSPBx [15] and pharmaceutical companies currently have no new antibiotics in the pipelines to replace oral FQs.

The necessity and cost-effectiveness of performing RS cultures in all men prior to TRUSPBx have been debated. Studies have demonstrated cost-savings of up to 4,499 USD per averted PTIC [18], but have also reported that anywhere between 27 and 45.7 men from the general population must be screened to prevent one PTIC [18,19,27]. However, a selected approach for men with known risk factors may have increased benefits [16], especially considering that results from previous cost-benefit reports are difficult to generalize. This is because the local prevalence of FQ-resistant bacteria will play a crucial role in the calculation of whether TAP is cost-efficient in a given region [28]. Moreover, the cost of medical care varies by region. Here, we clearly demonstrate the benefits of RS-guided TAP in reducing PTICs in our selected cohort of men at high-risk for infectious complications. Thus, in order to render TAP both effective and cost-efficient, the selection of patients who could benefit from TAP, via the identification of risk factors for developing PTICs, may be of major importance.

The most common risk factor for PTICs is exposure to antimicrobials within six months prior to biopsy [9]. Moreover, aside from known risk factors for PTICs including diabetes, immunosuppression, and urological factors [6], hospital employees and international travellers to areas where FQ resistance is endemic are also at increased risk [12,13]. A history of previous prostate biopsies is another risk factor, albeit highly debated, for PTICs [16,24]. In our MVA we found that recent antibiotic utilization, CCI≥1 and a history of UTIs/prostatitis before biopsy were the most powerful predictors for PTICs. These clinical conditions should thus be carefully considered, especially for patients at high risk for PTICs, in order to select patients who would benefit from TAP.

This study is, to date, the first to examine the impact of TAP in a selected cohort of men with well-known risk factors for developing PTICs; whereas previous studies on this topic have focused on the general population of men and have shown conflicting results. This study was conducted with a rigorous methodology while previous studies have shown various methodological limitations. Some previous studies examined only the prevalence of severe complications and ignored minor, more frequent, infectious complications or had a short follow up period, thus leading to an underreporting of PTICs [29]. In our study, rather, we assessed both major and minor PTICs and used a long follow up period. An inadequate registration method in previous studies could also have led to underreported infectious complications. The use of direct patient contact through a telephone call or a follow-up consultation is certainly more accurate than electronic medical records in the identification of PTICs [29].

Our study is not devoid of limitations. It was conducted with a relatively small, homogenous cohort of men and thus deserves external validation with an independent, larger and more diverse sample. Although the characteristics of the groups were similar, we cannot exclude unknown confounding factors. We did not perform a cost effectiveness analysis but we clearly demonstrated the significant impact of TAP in reducing PTICs in men with risk factors for infection after biopsy, identifying a specific group of patients who could most benefit from RS-guided prophylaxis. The retrospective nature of the study is another limitation. Also, the dipstick analysis performed on every patient to confirm nitrite negative and leukocyte esterase-negative urine cannot be considered as accurate as urine culture. However, current EAU guidelines did not recommend urine culture before TRUSBx [21]. Finally, as described above, patients may harbor multiple strains of both FQ-sensitive and FQ-resistant rectal flora, dictating consideration for repeated longitudinal screening [30].

## Conclusion

This cross-sectional study provides new clinically-relevant evidence that RS-guided prophylaxis significantly reduces PTICs compared to EAP in a cohort of men at high risk for infectious complications. Importantly, 23.5% of patients with RS culture results were found to have FQ-resistance E. coli. Moreover, recent antibiotic utilization, CCI≥1 and a history of UTIs/prostatitis before biopsy emerged as the most powerful predictors for PTICs. Overall, the current results indicate a clinical need for comprehensive investigations of potential risk factors for PTICs in order to select candidates who would benefit most form TAP, keeping in mind the extraordinary epidemiological and socio-economical impact of these infectious complications.

## Supporting Information

S1 Dataset Dataset containing data for statistical analyses. (XLS)
